# Segmentation of Myocardial Boundaries in Tagged Cardiac MRI Using Active Contours: A Gradient-Based Approach Integrating Texture Analysis

**DOI:** 10.1155/2009/983794

**Published:** 2009-06-11

**Authors:** Aymeric Histace, Bogdan Matuszewski, Yan Zhang

**Affiliations:** ^1^Equipe Traitement de l'Information et Système (ETIS), UMR CNRS 8051, 6 avenue du Ponceau, F-95000 Cergy, France; ^2^Applied Digital Signal and Image Processing Research Centre (ADSIP), University of Central Lancashire, Preston PR1 2HE, UK

## Abstract

The noninvasive assessment of cardiac function is of first importance for the diagnosis of cardiovascular diseases. Among all medical scanners only a few enables radiologists to evaluate the local cardiac motion. Tagged cardiac MRI is one of them. This protocol generates on Short-Axis (SA) sequences a dark grid which is deformed in accordance with the cardiac motion. Tracking the grid allows specialists a local estimation of cardiac geometrical parameters within myocardium. The work described in this paper aims to automate the myocardial contours detection in order to optimize the detection and the tracking of the grid of tags within myocardium. The method we have developed for endocardial and epicardial contours detection is based on the use of texture analysis and active contours models. Texture analysis allows us to define energy maps more efficient than those usually used in active contours methods where attractor is often based on gradient and which were useless in our case of study, for quality of tagged cardiac MRI is very poor.

## 1. Introduction

Non invasive assessment of the cardiac function is of major interest for the diagnosis and the treatment of cardiovascular pathologies. Whereas classical cardiac MRI only enables radiologists to measure anatomical and functional parameters of the myocardium (mass, volume, etc.), tagged cardiac MRI makes it possible to evaluate local intramyocardial displacements. For instance, this type of information can lead to a precise characterization of the myocardium viability after an infarction. Moreover, data concerning myocardium viability makes it possible to decide of the therapeutic medical treatment, angiopathy, or coronary surgery and following of the amelioration of the ventricular function after reperfusion.

**Figure 1:**
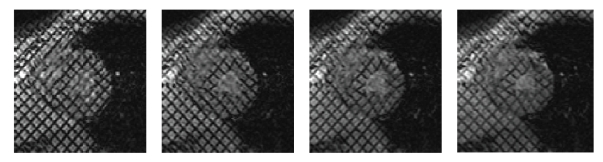
Short-Axis-Tagged MRI acquisition between end-diastole and end-systole.

The Space Modulation of Magnetization (SPAMM) acquisition protocol [1] we used for the tagging of MRI data, displays a deformable 45 degrees oriented dark grid which describes the contraction of myocardium (Figure 1) on the images of temporal Short-Axis (SA) sequences. Thus, the temporal tracking of the grid can enable radiologists to quantify cardiac geometrical parameters within myocardium. 

 Numerous studies were carried out concerning the analysis of the deformations of the grid of tags on SA sequences (See [2, 3] for reviews of these studies.). First part of them is based on a direct estimation of the displacement field of the myocardium [4–13], the other part on an indirect estimation of the displacement field [14–25].

**Figure 2:**
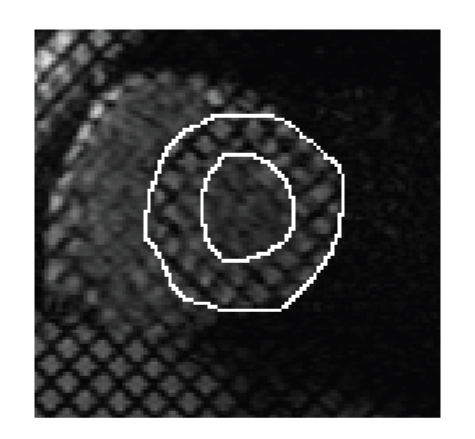
Manual detection of epicardial (external circle) and endocardial (internal circle) boundaries of the Left Ventricle (LV) on a Short-Axis-Tagged MRI acquisition.

A common step of all these approaches is the segmentation of myocardial boundaries for each instant of Left Ventricular (LV) contraction (diastole) (see Figure 2 for a manual segmentation of these boundaries) since LV contraction represents 80% of the whole heart contraction function. 

 This segmentation step is of primary importance since detection and tracking of the grids are made on this particular area for locally quantified LV displacements.

**Figure 3:**
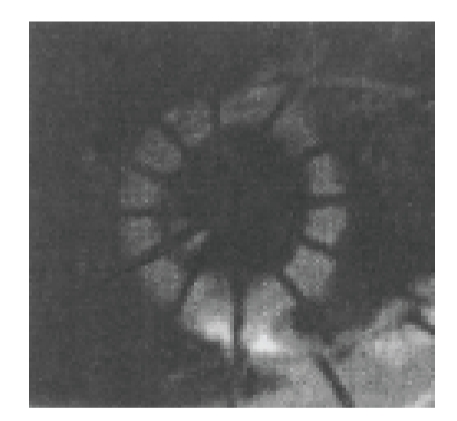
Radially tagged cardiac MRI taken from [26].

Among all previous cited papers, the only study integrating automatic detection of endocardial and epicardial boundaries within the tracking of the grid process was developed by Guttman [26] and carried out on radially-tagged acquisitions (Figure 3). 

 This method based on a prior erasure of tags using nonlinear filtering, turned out to be inappropriate to our images which are not radially tagged as one can notice on Figure 1. Moreover, this particular type of tagging is no more used in medical practice.

All other methods dealing with this segmentation problem involve manual detection of the myocardial boundaries [16, 27, 28], or a detection previously made on classical cardiac MRI sequences [2] or on filtered ones [3] and as such do not entirely address to the problem of in routine clinical practice.

In this article we present an alternative method for the automatic detection of myocardial boundaries on tagged cardiac MRI which integrates active contours and texture analysis. Our method enables an automatic detection of myocardial boundaries of LV on SA sequences and then an optimized tracking of the grid of tags within myocardium is possible.

Concerning the layout of this paper, next section is dedicated to the presentation of the global segmentation method of myocardial boundaries. Sections 3 and 4 deal with the computation of what we call energy maps thanks to texture analysis. Following section presents visual results of segmentation obtained on different patients and a statistical validation of the developed method. Last section is dedicated to discussion.

## 2. Active Contours and Context

Originally proposed in [29], active contours for segmentation have attracted extensive research in the past two decades. The basic idea of the active contour is to iteratively evolve an initial curve towards the boundaries of the target objects driven by the combination of internal forces determined by the geometry of the evolving curve and the external forces induced from the image.

Image segmentation methods using active contours are usually based on minimising functionals which are so defined that curves close to the target boundaries have small values. For instance, in [29], authors formerly proposed the following functional:

(1) $$\begin{array}{ll} E\left(C\right) & =\alpha \int_{0}^{1}‍{\left|C^{\prime }\left(q\right)\right|}^{2}dq+\beta \int_{0}^{1}‍{\left|C^{\prime \prime }\left(q\right)\right|}^{2}dq\\ & \;-\lambda \int_{0}^{1}‍\left|\nabla u_{0}\left(C\left(q\right)\right)\right|dq, \end{array}$$

where *C*(*q*) is a parameterized flat curve, *u*_0_ the initial image data and *α*, *β*, *λ* are positive constants. The first two parameters *α*, *β* control the regularity of the curve (*E*_intern_) and *λ* controls the attraction of the curve to the targeted boundaries (∇*u*_0_, with ∇ the classical gradient operator) of the studied image *u*_0_ (*E*_extern_). To solve these functional minimisation problems, a corresponding partial differential equation is constructed as the Gateaux derivative gradient flow resulting in a curve evolution.

To obtain interesting results with minimisation of (1), initialization of the curve has to be made close to the boundary of the structure to be segmented. This drawback is directly linked to the computation of the external energy induced from the image which is based on a classical gradient operator. As a consequence, if the initialization of the curve is made too far from the targeted structure, other local minima of the E_extern_ can corrupt final segmentation result.

However, in many applications of medical image segmentation, initialization of the curve has to be simple and fast and as result performed far from the target. For instance, initialization is often made on the boundary of the image, or near the center of gravity of a particular Region of Interest.

Taking this into consideration, we propose an evolution of the functional described by (1) given by

(2) $$\begin{array}{ll} E\left(C\right) & =\alpha \int_{0}^{1}‍{\left|C^{\prime }\left(q\right)\right|}^{2}dq+\beta \int_{0}^{1}‍{\left|C^{\prime \prime }\left(q\right)\right|}^{2}dq\\ & \;-\lambda \int_{0}^{1}‍\left|\nabla u_{\text{map}}\left(C\left(q\right)\right)\right|dq+\kappa \int_{0}^{1}‍n\left(C\left(q\right)\right)dq. \end{array}$$

As one can notice, two terms are added to the classical functional. The last one, *κ*∫_0_^1^ **n**(*C*(*q*))*dq* is a classical balloon energy formerly introduced in [30]. To explain its role, let us consider a circle as the initializing curve which evolution law is only driven by this energy with *κ* > 0. For each step of the evolution process, the circle has no other solution than to spread all along its local normals (**n**); the diameter of the circle grows up. It is an extra expanding term usually found necessary for quicker convergence. This energy has shown to be useful for fast growing of the curve when initialization is made far from the targeted boundary.

**Figure 4:**
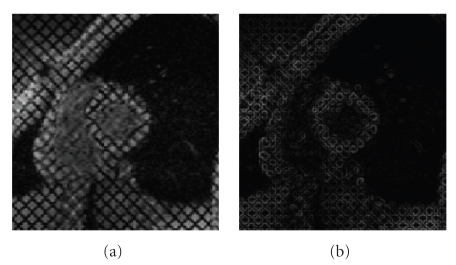
(a) Original image, (b) Norm of the corresponding gradient. As one can notice, the grid of tags does not allow us to obtain a good gradient attractor for myocardial contours.

The term given by *λ*∫_0_^1^|∇*u*_map_(*C*(*q*))|*dq* is derived from the former one of (1) and represents the induced boundary energy (*E*_extern_) adapted to our particular domain of application, tagged cardiac MRI. Indeed, the grid of tags does not allow us to obtain a good gradient attractor (Figure 4). As a consequence, the boundary-based energy is computed from a preprocessed version *u*_map_ of original image *u*_0_ which is described next section.

## 3. Computations of *u*_map_ Images

### 3.1. Endocardial Boundary

A major property observed on SA tagged MRI sequences is the fast erasure of tags in the cardiac cavity due to blood circulation (see Figure 1). To explain this phenomenon, one must understand that tagging process is obtained thanks to a saturation of hydrogen atoms in surfaces orthogonal to main orientations of the grid (see [1] for complete description of the process). As a consequence, muscles, fat tissues, and blood are tagged. But, considering cardiac motion, between two phases of contraction, blood is pumped out of the cardiac cavity into the main circulation. Therefore, tagged cells of blood are no more visible during acquisition process as soon as contraction has begun.

This property is of primary importance since image can be roughly divided into two areas: a tagged area (1) where the tracked grid remains visible and a homogenous area (2), roughly the cardiac cavity of Left and Right ventricles, where tagged are no more visible. As a consequence, areas (1) and (2) can be easily discriminated by simple texture parameters calculated on a local kernel like mean, and standard deviation. Area (1) is characterized by a standard deviation having bigger values than area (2) which is more homogenous (absence of tagging). For both areas, means remain nearly the same.

**Figure 5:**
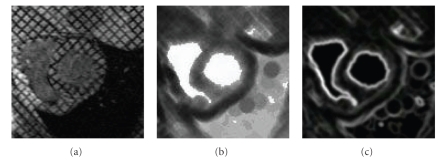
(a) Original image *u*_0_, (b) Computation of *u*_map_^endo^ with (*w*_*σ*_/*w*_*m*_) = 2 and *N* = 11, (c) ∇*u*_map_^endo^.

Considering this, we propose the calculation of a mean (M)-standard deviation (*σ*) image to build a precise gradient-based energy to detect the endocardial boundary (Figure 5(c)). This map is obtained by computation of (3) on the processed original sampled image *u*_0_(*i*, *j*) where (*i*, *j*) denotes the indices of a given pixel:

(3) $$u_{\text{map}}^{\text{endo}}\left(i,j\right)=w_{m}\cdot m_{N}\left(i,j\right)-w_{\sigma }\cdot \sigma_{N}\left(i,j\right).\;$$

*w*_*m*_ and *w*_*σ*_ are, respectively, the weights given to the mean computed on a kernel of size *N* ∗ *N* centered on the processed pixel and the weight given to the standard deviation computed with the same kernel. *w*_*m*_ and *w*_*σ*_ verify *w*_*m*_ + *w*_*σ*_ = 1.

 As one can notice on Figure 5(b), the computation of the mean-standard deviation image makes enhancement of the cardiac cavities of LV possible (pixels of high intensity). This image leads, on the one hand, to a possible automatic detection of the center of the LV cardiac cavity (that can be used for active contour initialization), and, on the other hand, to a gradient-based energy (Figure 5(c)) totally adapted to our purpose.

### 3.2. Epicardial Boundary

The gradient-based energy for the segmentation of the epicardial contour was more complex to compute since, as one can see on Figure 1, the boundary is hard to detect visually even for experts. Moreover, as for endocardial contour, active-contour segmentation cannot simply integrate tagged MRI gradient as attractor.

**Figure 6:**
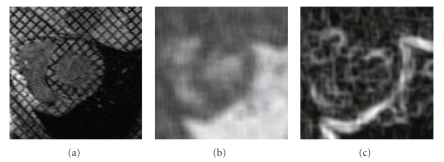
(a) Original image, (b) Computation of *u*_map_^epi^ with *N* = 5, (c) ∇*u*_map_^epi^.

As a consequence, to compute a useful *u*_map_^epi^, it appeared interesting to analyze the particular texture of the lung (dark area situated on the right of Figure 1). This area, compared with the rest of tagged MRI, is described by a rough texture. Thus, the use of second-order texture parameters and more particularly, the calculation of the co-occurrence matrix entropy on an *N* ∗ *N* block, can make the enhancement of the lung area possible by characterizing it with high entropy coefficients (Figure 6(b)). The map *u*_map_^epi^ then obtained allows us to compute an interesting gradient-based energy (Figure 6(c)). 

## 4. Practical Implementation

### 4.1. Computation

**Figure 7:**
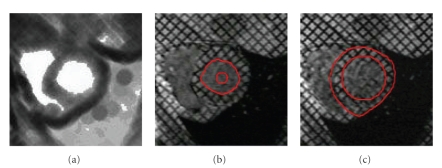
(a) Computation of *u*_map_^endo^ with (*w*_*σ*_/*w*_*m*_) = 2 and *N* = 11 for automatic localization of the barycenter of LV cavity, (b) initialization and detection of endocardial boundary, and (c) initialization and detection of epicardial boundary.

Practically speaking, in order to perform a quick computation, detection (on the first image of the sequence) and tracking of the myocardial boundaries (on the other images describing diastole) are made separately; the detection method is divided into five steps:

1. *u*_map_^endo^ is first computed and barycenter of the LV cavity is used for automatic active contour initialization (a circle) (Figures 7(a) and 7(b));
2. considering the given automatic initialization of step 1, a first fast growing (only taking into account the balloon force) of the active contour is used to obtain a rough segmentation of the endocardial contour;
3. the resulting curve from step 2 is then used as initialization for boundary-based evolution considering corresponding term of (2) with *u*_map_ = *u*_map_^endo^ (Figure 7(b));
4. since epicardial contour is situated close to the endocardial one (see Figure 2), an automatic radial spreading of the curve detected step 3 (no more than 4 pixels) is performed to obtain a rough segmentation of it;
5. the curve of step 4 is then used as initialization for boundary-based evolution considering corresponding term of (2) with *u*_map_ = *u*_map_^epi^ (Figure 7(c)).

Considering now tracking, in order to have a quick implementation, each detected myocardial boundaries (endocardial and epicardial) at instant *t* of the diastole is used as initialization for a boundary-based evolution at instant *t* + 1.

For epicardial contour detection and tracking, concerning part of the contour where gradient-based energy fails to bring good attraction (left part of the boundary), the coherence of the detection is obtained by setting to zero the *λ* parameter of (2). To do so, a test of distance (Euler) between the new calculated coordinates of the active contour point and the center of the LV cavity is computed. Indeed, compared to endocardial contour, global displacement of the epicardial one is still less important. As a consequence, if distance to the center of the new coordinates control point appears to be incoherent (to far from the precedent one), calculation is made again with *λ* = 0. Geometrical constrains are privileged to ensure coherence of the result.

### 4.2. Results

**Figure 8:**
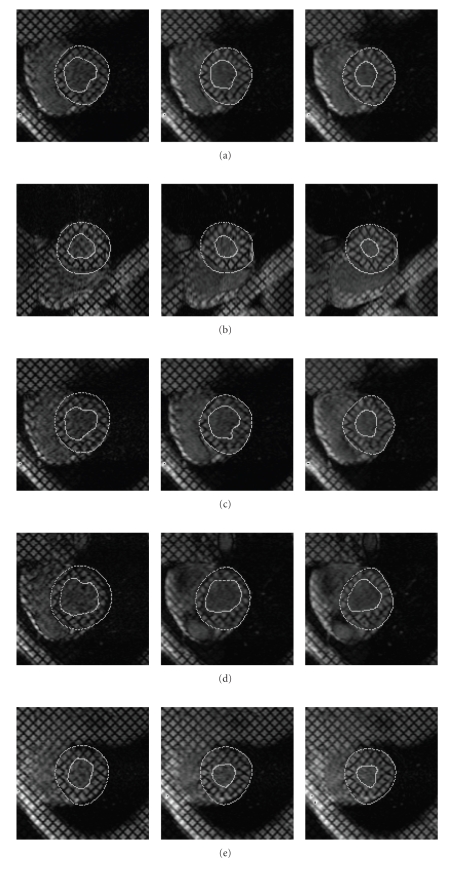
Detection and tracking of endocardial and epicardial boundaries on SA sequences all along diastole for 5 different patients.

Figure 8 shows results of detection for 5 different patients (extracted from a global set of 8). 

 Considering first intrinsic performances of the proposed method, as one can notice, the implemented method is robust as regard of the initialization which is always the same in each different case. Moreover the developed method is reproducible and does not need new tuning of the different parameters for new patients as Figure 8 shows where all segmentations have been made with same setting of the different parameters presented in previous section.

**Figure 9:**
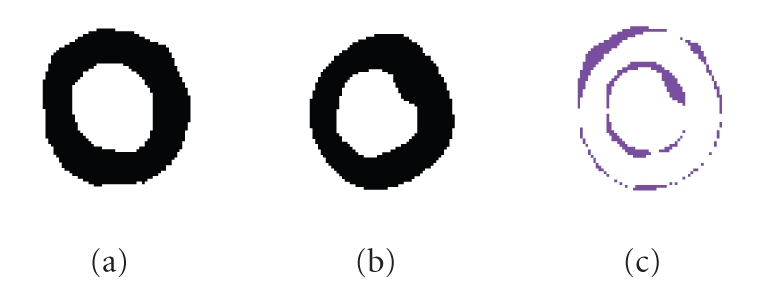
Detection mask obtained after segmentation of myocardial boundaries on a particular image: (a) expert's mask (manual segmentation), (b) automatically segmented mask (presented method), and (c) superposition of both (dark surface stands for mismatched pixels of both segmentation).

Considering now performances of the proposed approach in terms of precision, we propose a first statistical analysis made on a set of 8 patients as a basis for a future more complete analysis made on a larger scale. More precisely, for each of the 8 patients, 6 images extracted from a synchronized systole acquisition made at a median slice level of the LV are considered. For each image of a particular sequence, the semiautomatic segmentation of endocardial and epicardial boundaries of the LV and the corresponding myocardial surface is generated (see Figure 9(a)). The same study, starting from a manual expert segmentation of the myocardial boundaries, is also performed (see Figure 9(b)). For each image of a sequence, automatic and manual surfaces are compared (see Figure 9(c)) thanks to a calculation of the ratio corresponding to the matching and nonmatching pixels.

 More precisely, each pixel of the automatic generated mask is identified as being a True Positive (TP) pixel or a False Positive (FP) pixel or a True Negative (TN) pixel or at last a False Negative (FN) pixel. Table 1 shows the average number of each type of pixels (expressed in percentage of the average total number of pixels of each pixel class within manual generated myocardium surface) for each of the 6 considered time steps of the systole.

 Calculation of the ratio between matching pixels of both surfaces (TP) shows that for 48 processed images (6 per patients) more than 80% of the different surfaces are matching. Moreover, only more or less 3% of the automatic detected pixels are considered as FP, that is to say that they do not match at all expert surface. Those pixels are often situated near endocardial boundaries. This is above all due to the papillaries muscles of the LV which are also tagged and as a consequence influence expert segmentation (they tend to integrate them within the cardiac cavity). One can notice that in this case, the expert's segmentation of endocardial boundaries can lead to an overestimation of the surface area of LV's cavity since papillaries muscle must not be taken into account (which is the case with proposed automatic method).

No comparison with other methods are proposed since, as we mentioned it before, proposed automatic methods of the literature are not directly performed on tagged cardiac MRI but always on classical cardiac MRI (double acquisition).

## 5. Conclusion and Outlooks

In this article, we propose an automatic approach for segmentation of epicardial and endocardial boundaries of the LV directly on tagged cardiac MRI. The detection of the endocardial and epicardial boundaries on SA sequences is fully automatic and satisfactory, whereas the literature always involves manual detection during the analysis of tagged MR images. About the results of the detection of the epicardial contours, the method allows us to obtain satisfying results which are in agreement with medical specialists opinion. The method is less robust for endocardial segmentation, but still performs well. The way the method is initialized allows it not to be too dependant of this important step. The fact that even visually, detection is still very difficult for radiologists, and is particularly important for the consideration of our results.

As far as the robustness is concerned, progresses still to be made as the segmentation is dependent on the values the weights selected for the different energies and the size *N* of the neighborhood on which texture maps are computed.

Regarding precision of the method, we have presented a first statistical study which shows very promising results. If this statistical study still to be improved in order to characterize intra- and interoperator variabilities, obtained estimation allows us to go to a step further in the use of the proposed method. It is now possible to use these results (shared with those given by the tracking of the grids on SA) to develop a 2*D* + *T* analysis of the myocardium. The aim of this study will be the calculation of local cardiac quantitative parameters correlated to “gold standards ones” (like fraction ejection) in order to early reveal eventual pathologies like ischemia, for example, but also to characterize myocardial viability after reperfusion.

First results have been already obtained. Classical cardiac parameters on 10 SA sequences as radial, circumferential, longitudinal displacements, torsion, or deformations have been calculated. We present in Table 2 a comparison between our obtained results for the quantification of the radial displacements and two studied of the medical literature.

As one can notice, our results are comparable to those of the medical literature.

**Figure 10:**
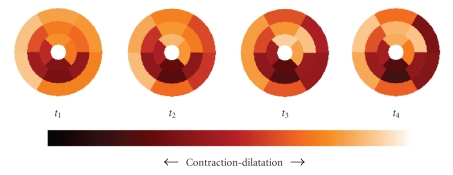
Radial contraction of the heart represented in accordance with the AHA's recommendations.

Moreover, it is also possible to realize a two-dimensional temporal map (according to the recommendations of the American Heart Association) characterizing the local displacements and local deformations of the myocardium (Figure 10). 

 Presented results could be interesting for radiologists to evaluate torsion, shearing, longitudinal, and radial displacements of the LV and then to draw early diagnoses of particular cardiopathies. These first results need now to be confirmed through a more complete clinical validation.

## Figures and Tables

**Table 1:** Average number of each type of pixels (expressed in percentage of the average total number of pixels of each pixel class within manual generated myocardium surface) for each of the 6 considered time steps of the systole.

*Systole Step*	Step 1	Step 2	Step 3	Step 4	Step 5	Step 6
TP	87.2%	85.1%	81.4%	79.7%	82.1%	81.0%
FP	3.1%	2.9%	2.2%	2.1%	1.9%	2.1%
TN	92.1%	93.1%	89.3%	90.1%	94.3%	91.8%
FN	1.2%	1.6%	1.9%	2.3%	1.7%	2.1%

**Table 2:** Comparison between our quantification and two studies of the medical literature concerning the estimation of the radial displacements (expressed in millimeters) for healthy volunteers.

	Base	Median	Apex
[31] (12 patients)	5.9 ± 0.4	6 ± 0.3	4.65 ± 0.2
[32] (31 patients)	5.0 ± 1.3	4.3 ± 1.1	4.2 ± 1.6
Our estimation (10 patients)	5.7 ± 0.5	4.9 ± 0.7	4.3 ± 0.9
